# Supplementary material for: Programmable responsive hydrogels inspired by classical conditioning algorithm
Source: Nat Commun. 2019 Jul 22;10:3267. doi: 10.1038/s41467-019-11260-3 (PMC6646376; doi:10.1038/s41467-019-11260-3)
Supplement: Supplementary file 1 — Supplementary Information [file 41467_2019_11260_MOESM1_ESM.pdf]

Supplementary Information for

Programmable responsive hydrogels inspired by classical conditioning algorithm

Zhang et al.

This PDF file includes:

Supplementary Methods
Supplementary Figures
Supplementary References

Supplementary Methods

Materials

The following chemicals were purchased from Sigma-Aldrich: gold(III) chloride trihydrate ($HAuCl4·3H2O$, >99.9% trace metals basis), sodium citrate tribasic dihydrate (BioUltra, 99.5%), tannic acid (TA, ACS reagent), potassium carbonate (K_2CO_3, 99.995% trace metals basis), potassium phosphate tribasic (K_3PO_4, 98%), silver nitrate ($AgNO_3$, 99.9999% trace metals basis), methyl formate (anhydrous, 99%), 1,3-propanesultone (>99%), 2,3,3-trimethylindolenine (98%), 2-hydroxybenzaldehyde (>99.0%), (±)-α-lipoic acid (>99%), sodium chloride (NaCl, BioXtra, >99.5%), sodium hydroxide (NaOH, 98%), pH 4 buffer solution (citric acid/sodium hydroxide/hydrogen chloride, Certipur), agarose (ultra-low gelling temperature, SKU: A5030), urea (powder, BioReagent), urease from *Canavalia ensiformis* (1 U mg^{-1}).

Hydrochloric acid (HCl) solution 1M (NIST standard solution) was purchased from Fisher Scientific. Ethanol (99.5%) was purchased from ALTIA Oyj (Finland). MilliQ water (18.2 MΩ, DirectQ 3 UV, Millipore) was used in all experiments.

Methods

Gold nanoparticles:

To synthesize gold nanoparticle with an average diameter of 10 nm and 13 nm, the seeded growth method in the presence of tannic acid was used[1]. All solutions were freshly prepared before the reaction. Briefly, 150 mL of 2.2 mM aqueous solution of trisodium citrate dihydrate was mixed with 0.1 mL of 2.5 mM tannic acid and 1 mL of 150 mM potassium carbonate in a 250 mL three-neck flask. The solution was heated to 70 °C in an oil bath. Subsequently, 1 mL of 25 mM $HAuCl_4·3H_2O$ solution was quickly injected under vigorous stirring, and the solution was kept at 70 °C for another 5 min. In this way, gold seeds with an average diameter of 3.5 nm were prepared. Directly after the formation of gold seeds, 55 mL of this solution was extracted, and 55 mL of 2.2 mM citrate solution was added. After the temperature of the solution reached again 70 °C, 0.5 mL of 25 mM $HAuCl_4·3H_2O$ solution was injected to initiate the growth of the seed. After 10 min, an identical injection was carried out. This growth procedure including extraction, addition of citrate and two injections of gold precursor was repeated for 4 times in total for 10 nm AuNPs and 6 times for 13 nm AuNPs.

To synthesize gold nanoparticles with an average diameter of 16 nm, the classical citrate reduction method was used[2]. Briefly, 100 mL of freshly prepared 0.01 wt% aqueous solution of $HAuCl_4·3H_2O$ was brought into boiling under reflux in an oil bath. Then 3 mL of 1.0 wt% trisodium citrate dihydrate solution was quickly injected under vigorous stirring. The solution was further refluxed for 10 min under stirring and left cooling at room temperature.

To synthesize gold nanoparticles with an average diameter of 20 nm and 43 nm, the citrate reduction method in the presence of silver nitrate was used[3]. All stock solutions were prepared freshly before synthesis. Briefly, to certain volume of 1.0 wt% citrate aqueous solution (1.1 mL for 20 nm and 0.3 mL for 43 nm), 1 mL of 0.5 wt% $HAuCl_4$ aqueous solution and 42.5 µL of 0.1 wt% $AgNO_3$ aqueous solution were added consecutively under stirring. Water was then added to bring the volume of the mixture solution to 2.5 mL, which was further stirred for 5 min. Note that the sequence of mixing and the duration of incubation are important. The solution was then quickly injected into 47.5 mL of boiling water under reflux in an oil bath. The solution was further refluxed for 1 h to ensure complete consumption of gold precursor.

Characterization of the AuNPs was carried out by transmission electron microscope (Tecnai 12). The size of the particles was analyzed using the "Analyze Particle" function of ImageJ (version 1.51s). The diameter was calculated from the area of the particles assuming that the particles are perfect spheres. More than 150 particles were analyzed for each sample.

For modification of the citrate protected AuNPs, lipoic acid was first dissolved in ethanol to make a 10 mM solution. 20 µL of 1M NaOH solution was added to 10 mL of the AuNP solution to adjust the pH of the solution to ~11. Then 1 mL of the freshly prepared lipoic acid solution was added dropwise under stirring. The solution was then shaken overnight on an orbital shaker. Subsequently, the solution was centrifuged at 15 °C for three times (30 min, Avanti J-26XP centrifuge, Beckman Coulter), each time with removal of the supernatant after centrifugation, dilution with water to 20 mL and addition of 20 µL of 1 M NaOH solution. The centrifugation conditions are: 30,000 g, 24,000 g, 22,000 g, 16,000 g, and 4,000 g for 10 nm, 13 nm, 16 nm, 20 nm, and 43 nm AuNPs respectively. After the last centrifugation, the sediment was collected and diluted with water to a final volume of 1 mL. The modified AuNPs were always prepared freshly before use.

Photoacid:

The photoacid was synthesized according to literature[4,5]. Briefly, 2,3,3- trimethylindolenine (3.18 g, 20 mmol) was mixed with 1,3-propanesultone (2.44 g, 20 mmol) and then heated to 90 °C for 6 h under nitrogen protection. The product was collected as a purple solid by filtration and washed thoroughly with diethyl ether. 600 mg (2.14 mmol) of the dried product and 292 mg of 2-hydroxybenzaldehyde (2.4 mmol) were then added to 12.0 mL of anhydrous ethanol, and the solution was refluxed overnight. The resulting orange solid was collected by filtration and rinsed thoroughly with ethanol (5 × 10 mL). The final product was dried in vacuum and stored in freezer (-20 °C) under nitrogen protection. The pH of the photoacid solution was measured by a Mettler Toledo SevenExcellence pH meter.

Hydrogel containing AuNPs and photoacid:

The composition of the gel was optimized for strong spectral change under mild conditions (Supplementary Fig. 2 – 6). The melting point of the agarose gel was determined by inverting the cuvette after incubation at different temperatures (Supplementary Fig. 7). To prepare the gel, 10 mL of water was added to 30 mg of agarose (for 0.3 wt% gel) in a pre-rinsed glass vial. The mixture was then heated to 70 °C and vortexed until the agarose was fully dissolved. After the clear agarose solution cooled down to room temperature, 0.78 mg of photoacid was added. The solution was then sonicated at 35 °C until complete dissolution of the photoacid. Subsequently, 1.92 mL of this solution was mixed with certain amount of 1 M NaCl solution (30 µL for the gels in Fig. 1, and 40 µL for the gels in Fig. 4 for faster self-assembly) in a disposable cuvette (BRAND, Sigma-Aldrich). Finally, 35 µL of the lipoic acid modified AuNP solution was added and mixed thoroughly. The cuvette was sealed by parafilm and stored in fridge (4 °C) overnight for gelation. The reference gel was prepared in the same way except that water was added instead of AuNP solution. For Fig. 4a-c, additional 1 M urea solution and 1 mg mL^{-1} urease from *Canavalia ensiformis* solution was added so that the final concentration is 20 mM for urea and 5 µg mL^{-1} for urease. This gel was conditioned directly without storage overnight. For Fig. 4d-f, certain amount of 1 M K_3PO_4 solution and liquid methyl formate were injected after association so that the final concentration after mixing is 20 mM for K_3PO_4 and 240 mM for methyl formate.

For the association process, the gel was irradiated at 50 °C in a water bath for 60 min (Fig. 1). Unless otherwise mentioned, the irradiation for the gel always consists of a 635 nm laser (140 mW cm^{-2}, RLTMRL-635-1W-5, Roithner Lasertechnik) and a collimated 455 nm LED (25 mW cm^{-2}, M455L3-C2, Thorlab). The intensity of the LED light was determined according to the minimal required intensity to fully activate the photoacid at 50 °C (Supplementary Fig. 1). Finally the cuvette was stored in fridge (4 °C) overnight for re-gelation. The UV-Vis spectra were measured on an Agilent Cary 5000 UV-Vis spectrometer. As reference, pure agarose gels were used, either in the gel or the sol state depending on the state of the sample.

The photothermal heating was done by irradiating the cuvette in air (635 nm laser + 455 nm LED). The temperature of the cuvette was recorded simultaneously by an infrared camera (T62101, FLIR). After irradiation, the sample was left undisturbed for 10 minutes to ensure comparable coloration of the sample (color recovery of the photoacid). Afterwards the cuvette was carefully inverted for photographing with a digital camera (Canon EOS 5D Mark III, EF 100 mm f/2.8L).

Rheological measurement of the agarose gel:

Rheological measurements were carried out using Anton Paar MCR 302, a dynamic rotational rheometer equipped with a Peltier plate (P-PTD 200), a Peltier hood (H-PTD 200), 50 mm cone-and-plate geometry (CP50-1) with 0.984° cone angle and raw waveform module, and RheoCompass software. Water droplets were added under the Peltier hood, next to the sample in order to prevent evaporation during the measurement. The rheological measurements were performed triplicated.

Frequency sweeps were performed at 0.15 Pa stress amplitude at both 20 °C and at 50 °C. Time sweeps were performed at 0.15 Pa stress and at an angular frequency 1.0 rad s^{-1}. The 0.15 Pa stress amplitude was chosen based on the preliminary stress amplitude sweeps (0.01 - 1 Pa) at both 20 °C and 50 °C. The raw waveform signal was good and steady between 0.05 - 1 Pa stress amplitudes (not shown here). After pipetting the sample onto the plate at 20 °C and after heating samples to 50 °C, they were allowed to equilibrate for approximately 10 minutes, which was observed as a steady flat curve during the time sweep (not shown here).

Supplementary Figures

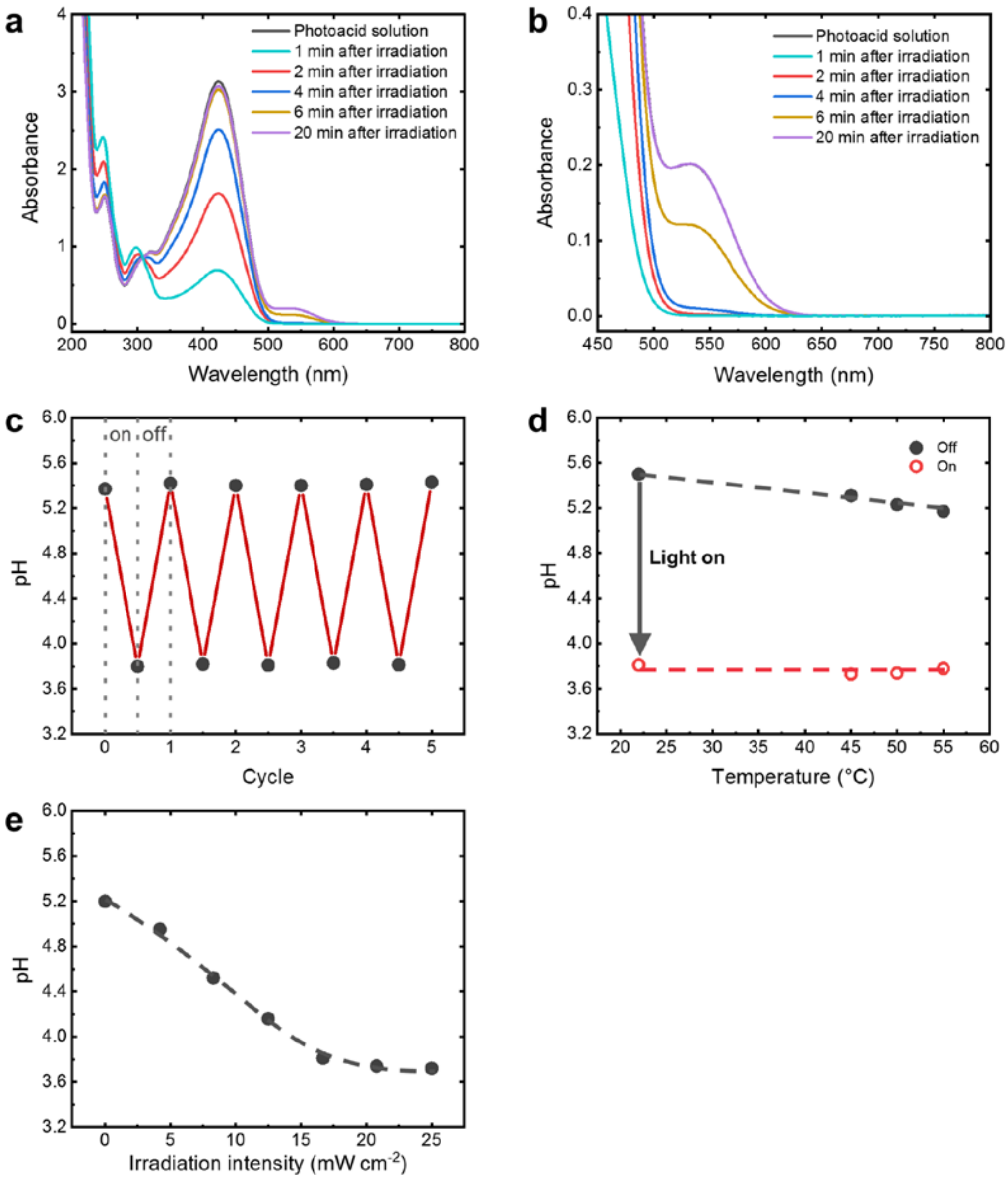

Supplementary Figure 1 | Light-triggered responses of photoacid. (a) UV-Vis spectra of the photoacid solution showing recovery after irradiation. **(b)** Enlarged view of **(a)**. The black curve is not visible due to complete overlap with the purple curve (20 min after irradiation). **(c)** pH change of a 0.2 mM photoacid solution upon irradiation and recovery in 5 cycles at room temperature. The irradiation was carried out for 5 min, followed by 20 minutes of recovery in darkness. Irradiation: 25 mW cm^{-2} at 455 nm. **(d)** pH change of a 0.2 mM photoacid solution at different temperatures upon irradiation. The temperature of the solution was controlled by a water bath, which was heated stepwise from 22 °C to 55 °C. The solution was equilibrated for 5 min before irradiation. The duration of irradiation and recovery was 5 min and 20 min respectively. The irradiation intensity was 25 mW cm^{-2} at 455 nm. Dashed lines are to guide the eye. **(e)** pH of the 0.2 mM photoacid solution at 50 °C under different irradiation intensities. The pH was measured after 5 min of irradiation at each intensity. Dashed line is to guide the eye.

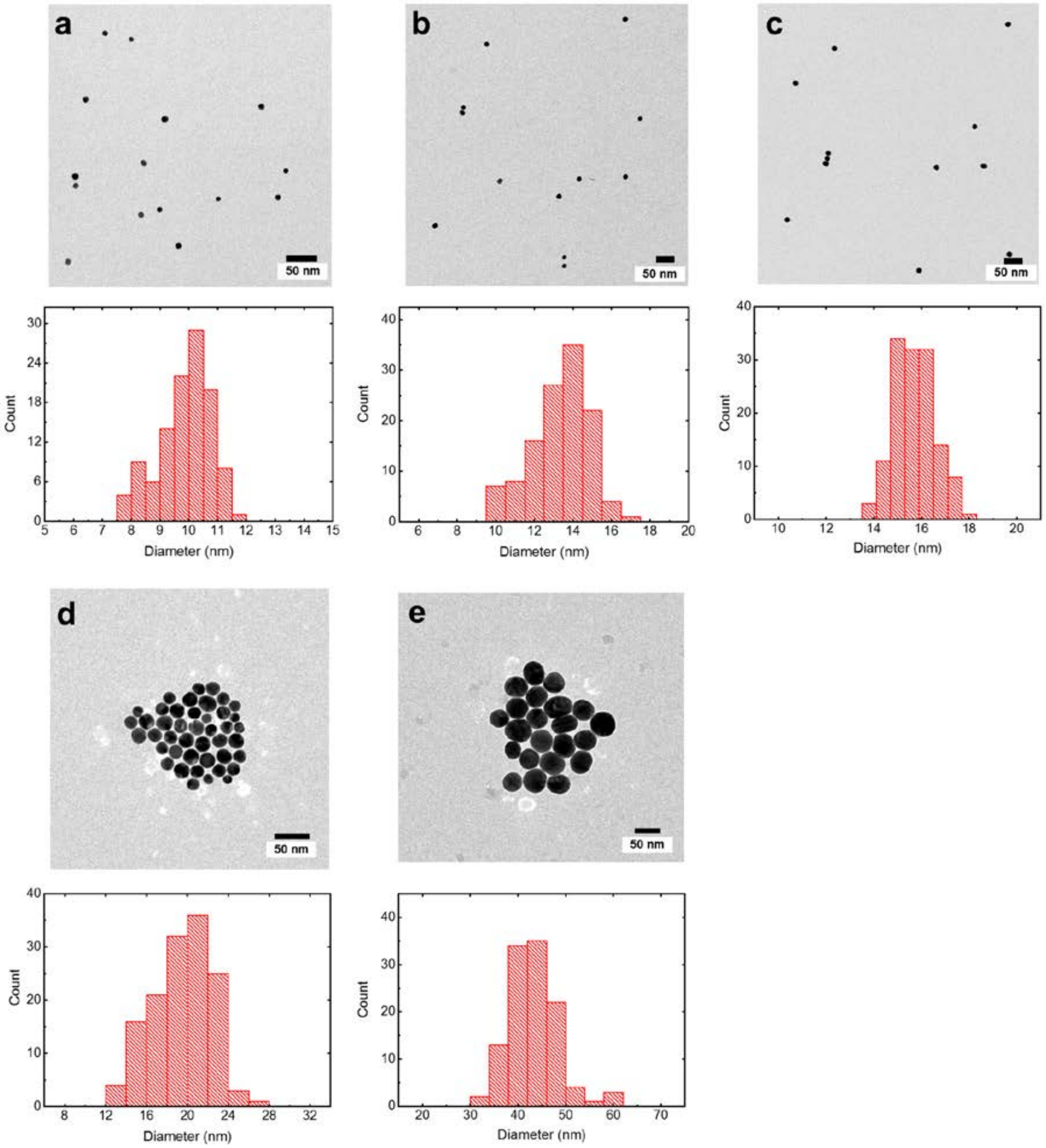

Supplementary Figure 2 | Representative TEM images of different AuNPs and the corresponding size distributions (lower panel). Particle size: **(a)** 10 nm (10.0 ± 0.9 nm), **(b)** 13 nm (13.3 ± 1.5 nm), **(c)** 16 nm (15.7 ± 0.8 nm), **(d)** 20 nm (19.8 ± 2.9 nm), and **(e)** 43 nm (43.1 ± 5.1 nm).

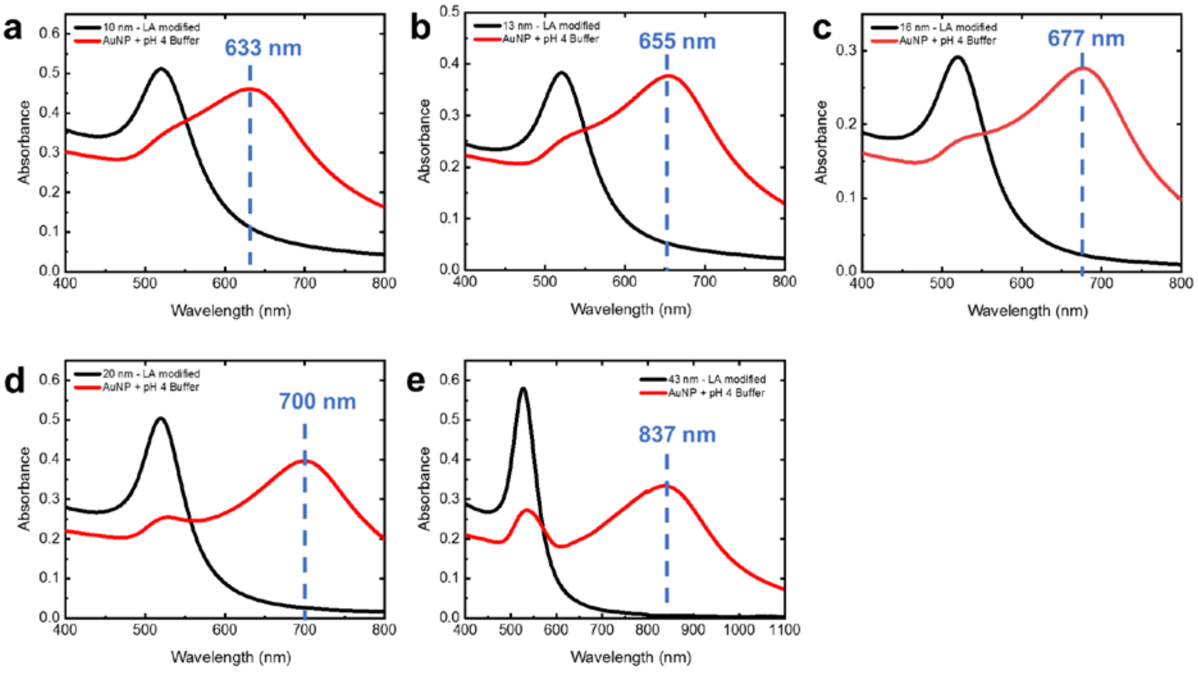

Supplementary Figure 3 | UV-Vis spectra of lipoic acid-modified AuNPs in solution before and after adding pH 4 buffer solutions. (a) 10 nm AuNPs, **(b)** 13 nm AuNPs, **(c)** 16 nm AuNPs, **(d)** 20 nm AuNPs, and **(e)** 43 nm AuNPs. Dashed lines indicate the position of the absorbance maximum after incubation. The buffer solution was added at a volume ratio of 1:19 (i.e., 20 × dilution of the buffer, final pH of the solution ~ 4.2), and the spectra was taken after incubation for 60 min.

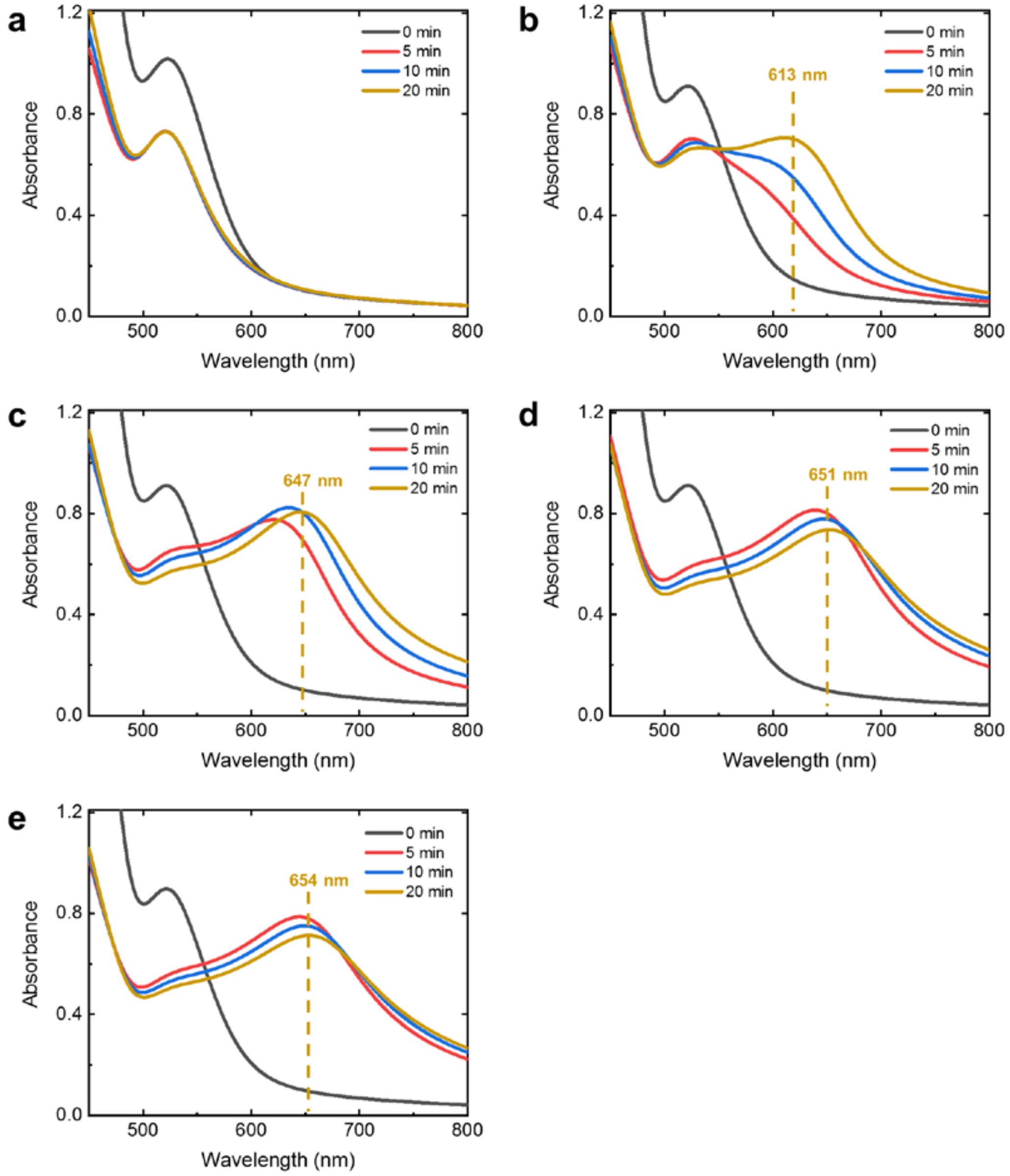

Supplementary Figure 4 | UV-Vis spectra of 13 nm AuNPs in 0.2 mM photoacid solution at different salt concentrations upon irradiation. **(a)** 0 mM NaCl, **(b)** 5 mM NaCl, **(c)** 10 mM NaCl, **(d)** 15 mM NaCl, and **(e)** 20 mM NaCl. The black curves show the original spectra of the solution (0 min). The irradiation was 455 nm LED (25 mW cm^{-2}). The spectra were taken immediately after the solution has been irradiated for a certain duration. The position of the maximum of the plasmonic band after 20 min irradiation is indicated by dashed lines.

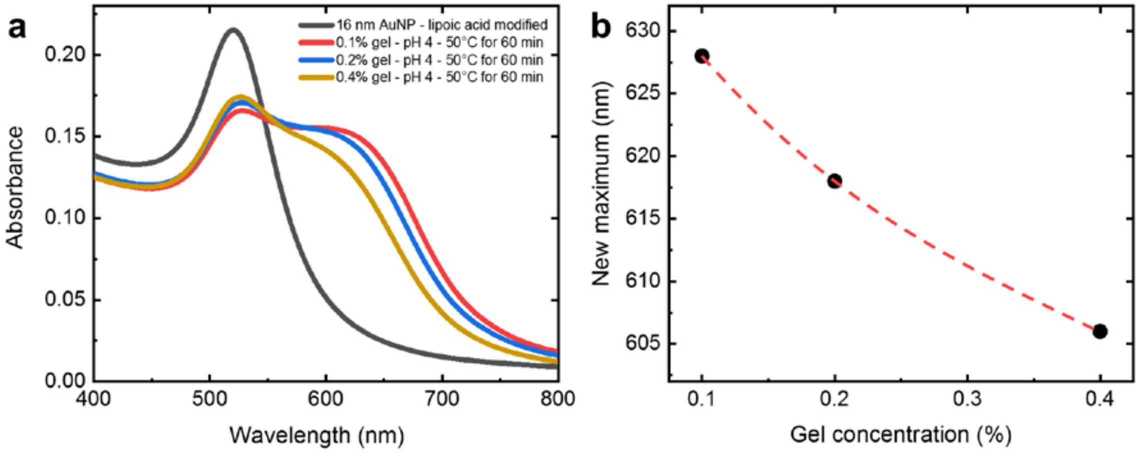

Supplementary Figure 5 | Influence of gel composition on the self-assembly of 16 nm AuNPs. (a) UV-Vis spectra of the gel after incubated at 50 °C for 60 min. pH 4 buffer was added at a volume ratio of 1:19 before the incubation (20 × dilution). **(b)** The position of the new plasmonic band maximum depending on the gel concentration shown in (A). Dashed line is to guide the eye. The 0.3 wt% was chosen as the 0.1 wt% and 0.2 wt% solutions did not form proper gels.

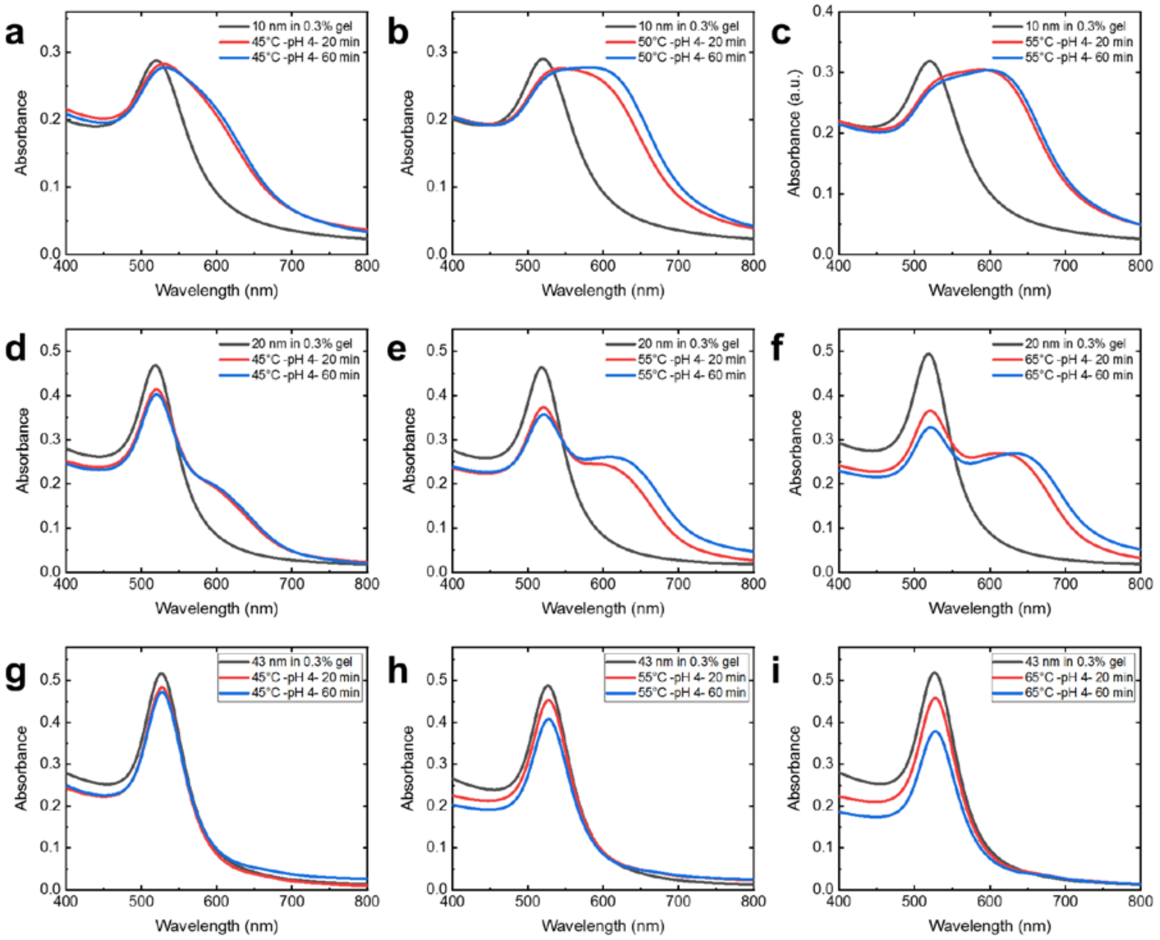

Supplementary Figure 6 | Screening of AuNP size and temperature for association. (a) – (c) UV-Vis spectra of 10 nm AuNPs in agarose gel at 45°C, 50°C and 55°C upon addition of pH 4 buffer (20 × dilution). **(d) – (f)** UV-Vis spectra of 20 nm AuNPs in agarose gel at 45 °C, 55 °C and 65 °C upon addition of pH 4 buffer (20 × dilution). **(g) – (i)** UV-Vis spectra of 43 nm AuNPs in agarose gel at 45 °C, 55 °C and 65 °C after addition of pH 4 buffer (20 × dilution). The gels containing AuNPs were immersed in water bath at different temperature for 5 min, after which 5 vol% of pH 4 buffer was added. The spectra were taken after incubation for indicated duration.

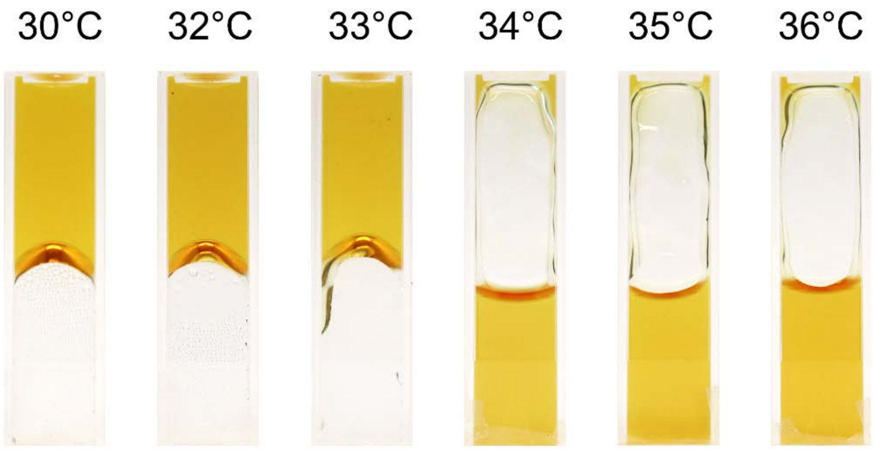

Supplementary Figure 7 | Photographs of the agarose gels containing AuNPs and photoacid in inverted cuvettes after incubation for 30 min at different temperatures. After preparation, the gels were melted at 60 °C and then re-gelled overnight to mimic the association process. Afterwards the gels were immersed in water baths at different temperatures and incubated for 30 min. Note that at 33 °C the gel already showed sign of melting, i.e. droplet on the wall.

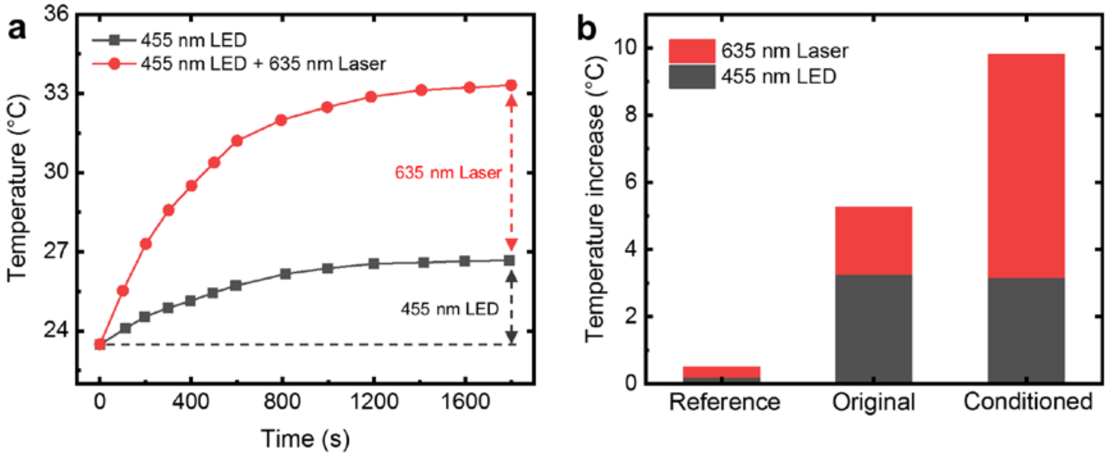

Supplementary Figure 8 | Photothermal heating on gels. (a) Temperature change of the conditioned gel upon irradiation. (455 nm LED and 455 nm LED + 635 nm Laser). **(b)** Temperature increase after 30 min irradiation for reference gel (gel containing photoacid without AuNPs), original gel, and gel after association.

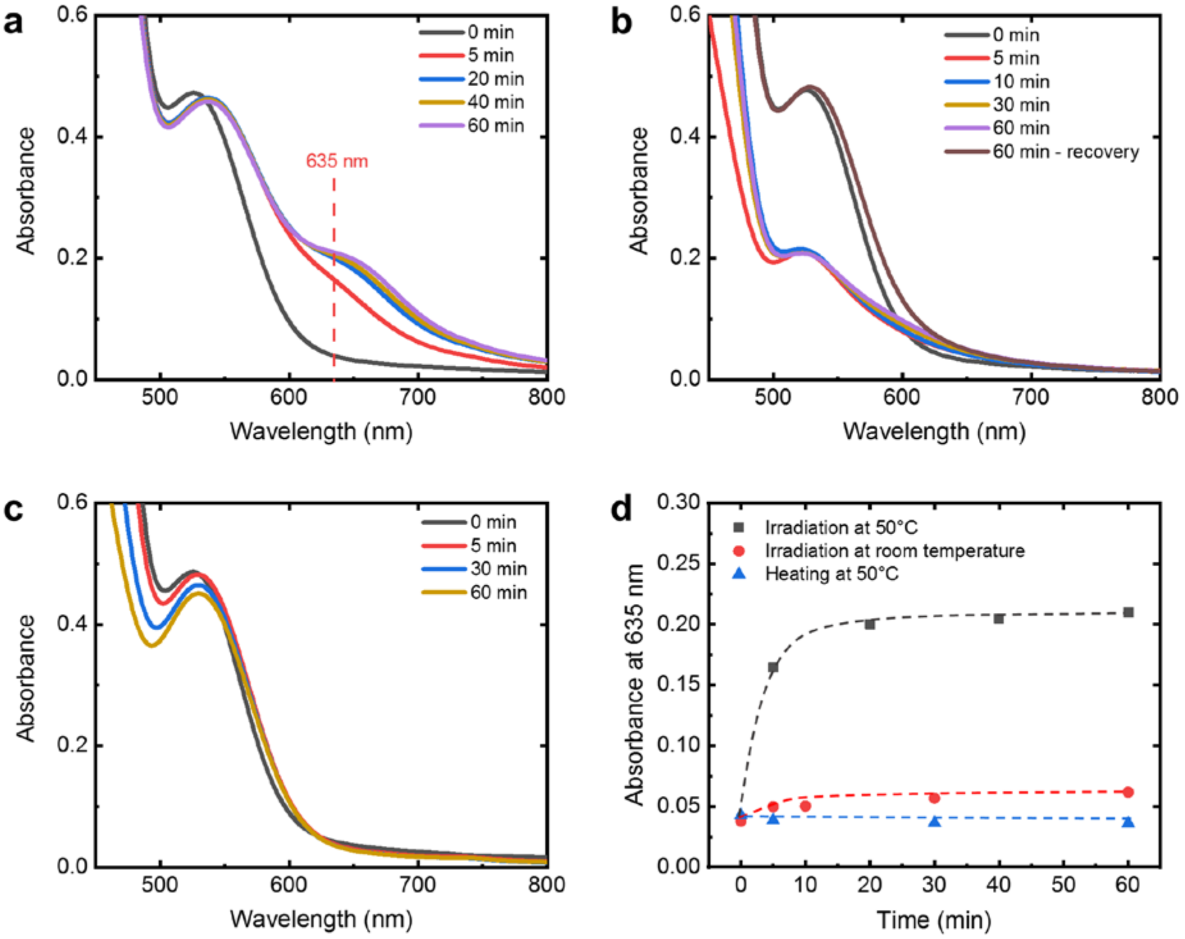

Supplementary Figure 9 | Kinetics of the association process. (a) UV-Vis spectra of the hydrogel during association. The position of 635 nm is marked by dashed line. **(b)** UV-Vis spectra of the hydrogel under irradiation at room temperature. The spectra were taken immediately after the irradiation for certain duration. The recovery curve was measured 20 min after the irradiation was switched off. **(c)** UV-Vis spectra of the hydrogel upon heating at 50 °C without irradiation. **(d)** Change of absorbance at 635 nm for gel during association (irradiation at 50 °C), under irradiation at room temperature, and upon heating at 50 °C. Dashed lines are to guide the eye.

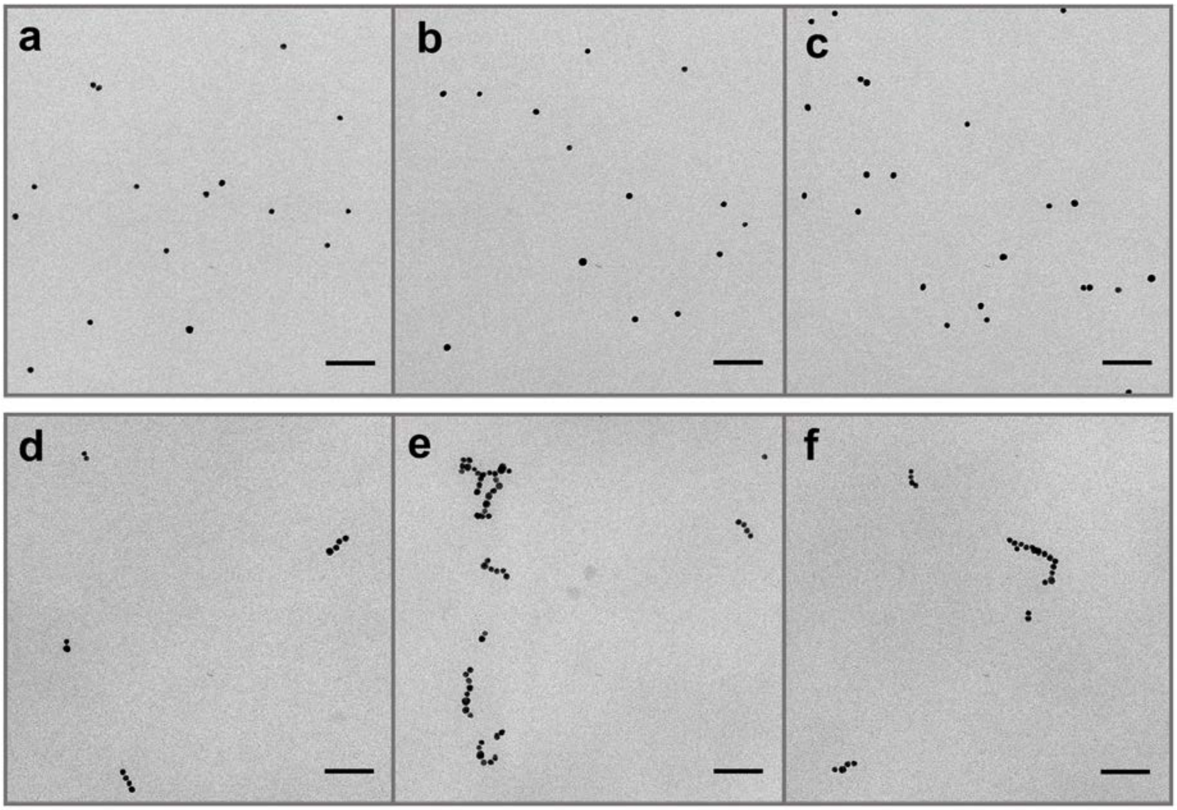

Supplementary Figure 10 | Representative TEM images of AuNPs in gels. (a) - (c) AuNPs in hydrogel after irradiation for 60 min at room temperature. **(d) - (f)** Hydrogel after association. Samples were prepared by pipetting 3 µL of the gels onto a TEM grid and removing the excess of the liquid. Scale bar: 100 nm.

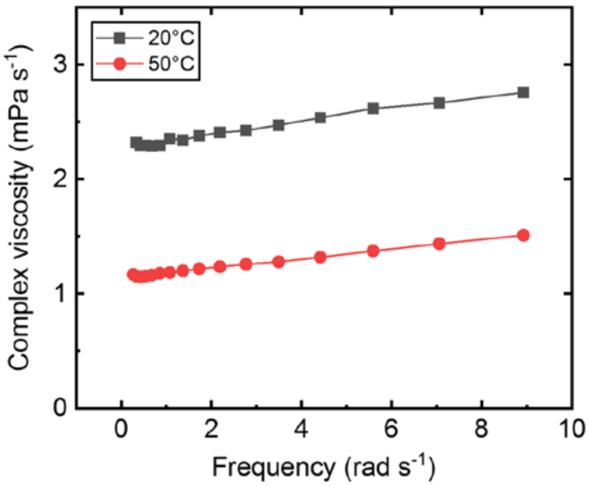

Supplementary Figure 11 | Temperature-dependent viscosity of the 0.3 wt% agarose in liquid state.

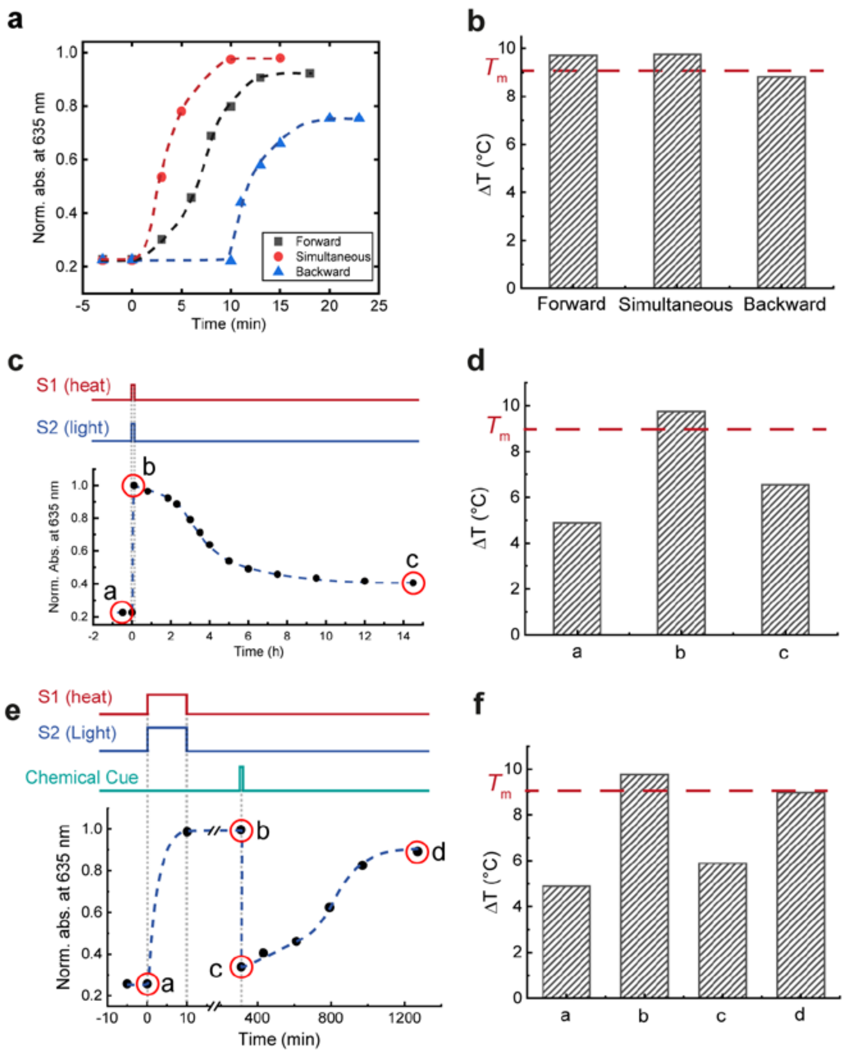

Supplementary Figure 12 | Temperature increase of the hydrogels upon irradiation. (a) Normalized absorbance at 635 nm of forward, simultaneous, and backward association. **(b)** Photothermal temperature increase of the gels after forward, simultaneous, and backward association. **(c)** Normalized absorbance at 635 nm during the forgetting process. The gel contains urea/urease. **(d)** Photothermal temperature increase of the gels at different points during the forgetting process, indicated in **(c),** as a, b, and c. **(e)** Normalized absorbance at 635 nm during the extinction and recovery process. The chemical cue is a mixture of K_3PO_4 buffer solution and methyl formate. **(f)** Photothermal temperature increase of the gels at different points during the forgetting process, indicated in **(e)** as a, b, c, and d. Melting point is indicated as T_m. Dashed lines are to guide the eye. Irradiation: 635 nm laser (140 mW cm^{-2}) and 455 nm LED (25 mW cm^{-2}). Duration of irradiation: 30 min.

Supplementary References

1. Piella, J., Bastús, N. G. & Puntes, V. Size-controlled synthesis of sub-10-nanometer citrate-stabilized gold nanoparticles and related optical properties. *Chem. Mater.* **28,** 1066–1075 (2016).

2. Frens, G. Controlled nucleation for the regulation of the particle size in monodisperse gold suspensions. *Nat. Phys. Sci.* **241,** 20–22 (1973).

3. Xia, H., Bai, S., Hartmann, J. & Wang, D. Synthesis of monodisperse quasi-spherical gold nanoparticles in water via silver(I)-assisted citrate reduction. *Langmuir* **26,** 3585–3589 (2010).

4. Shi, Z., Peng, P., Strohecker, D. & Liao, Y. Long-lived photoacid based upon a photochromic reaction. *J. Am. Chem. Soc.* **133,** 14699–14703 (2011).

5. Samanta, D. & Klajn, R. Aqueous light-controlled self-assembly of nanoparticles. *Adv. Opt. Mater.* **4,** 1373–1377 (2016).
